# Disrupting Dimeric β-Amyloid by Electric Fields

**DOI:** 10.1021/acsphyschemau.3c00021

**Published:** 2023-07-12

**Authors:** Pablo Andrés Vargas-Rosales, Alessio D’Addio, Yang Zhang, Amedeo Caflisch

**Affiliations:** Department of Biochemistry, University of Zurich, CH-8057 Zürich, Switzerland

**Keywords:** Alzheimer’s disease, molecular dynamics, electric fields, AlphaFold, SAPPHIRE, disordered proteins, secondary structure

## Abstract

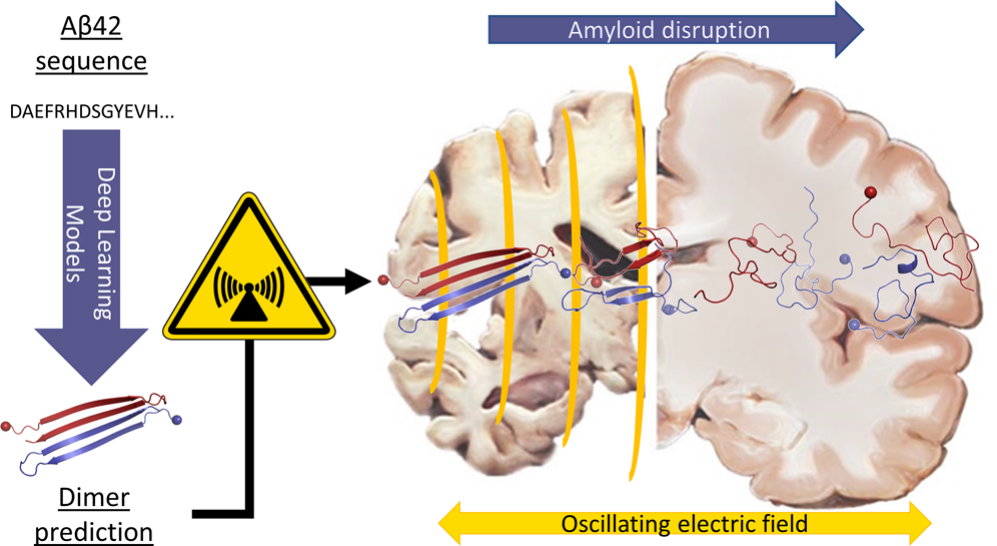

The early oligomers of the amyloid Aβ peptide are implicated in Alzheimer’s disease, but their transient nature complicates the characterization of their structure and toxicity. Here, we investigate the stability of the minimal toxic species, i.e., β-amyloid dimers, in the presence of an oscillating electric field. We first use deep learning (AlphaFold-multimer) for generating initial models of Aβ42 dimers. The flexibility and secondary structure content of the models are then analyzed by multiple runs of molecular dynamics (MD). Structurally stable models are similar to ensemble representatives from microsecond-long MD sampling. Finally, we employ the validated model as the starting structure of MD simulations in the presence of an external oscillating electric field and observe a fast decay of β-sheet content at high field strengths. Control simulations using the helical dimer of the 42-residue leucine zipper peptide show higher structural stability than the Aβ42 dimer. The simulation results provide evidence that an external electric field (oscillating at 1 GHz) can disrupt amyloid oligomers which should be further investigated by experiments with brain organoids *in vitro* and eventually *in vivo*.

## 1 Introduction

Alzheimer’s disease (AD) is the most frequent threat to the mental health of the elderly. At the molecular level, the self-assembly of the amyloid-β peptide (Aβ) impairs the structure and function of the neurons. The final products of the aggregation process are the amyloid plaques which consist of fibrils of the 42-residue (and/or 40-residue) Aβ peptide.^1^ Nevertheless, the evidence for the toxicity of plaques has been put into question,^2,3^ and there is substantial evidence for the role of early oligomers in the progression of AD.^4−6^ The minimal oligomers are Aβ dimers, which are transient and can aggregate into stable protofibrils.^7^ Cellular assays and *in vivo* experiments have revealed that Aβ dimers contribute to synapse dysfunction by perturbing glutamatergic transmission, and disrupt the memory of learned behaviors in rodents.^8,9^ Experimental data and kinetic modeling have shown that the transient oligomers of Aβ42 are particularly stable and productive in their conversion to fibrils, even in comparison to other aggregating peptides. These qualities make the oligomers interesting therapeutic targets under either the oligomer or fibril theories of Aβ42 toxicity.^10,11^

The pathways and kinetics of amyloidogenic peptides are difficult to monitor at the atomic level of detail by experimental means. Molecular dynamics (MD) simulations have shed light on the early aggregates despite the approximations inherent to the force fields and the short time scales accessible by atomistic models.^12−14^ The most populated conformations of the Aβ42 homodimer have been recently predicted by the use of atomistic MD simulations.^15^ First, an equilibrated structure of the monomer was sampled by microsecond-range MD simulations, after which two representative structures of the monomer were chosen for simulations of the dimeric system. An initial 1 μs simulation was performed, from which five subsequent 1 μs simulations were randomly restarted for a total of 6 μs sampling. This analysis has shown the propensity of the Aβ42 dimers to form β-strand hairpins in solution.^15^ Other groups have used different techniques to predict the ensemble of conformations of the dimer. One of such approaches is the use of blockwise excursion sampling, which has yielded dimers with a secondary structure content in agreement with circular dichroism (CD) experimental data.^16^ Furthermore the dimers were shown to consist mostly of turns and coils, with no highly populated cluster containing the hairpins, though propensity of β-strands was observed to be high in the C-terminal regions. Nonetheless, previous experimental results supported by MD simulations explored the importance of β-sheet-based planar dimers for the formation of stable fibrils.^17^ An important factor to consider in the simulation of intrinsically disordered proteins (IDPs) is the choice of force field used.^18^ The simulation results of Dehabadi and Firouzi provide evidence that CHARMM36m^19^ is an appropriate force field for the simulation of Aβ42 dimers.^16^

Deep learning (DL) has made a significant impact on the field of protein structure prediction by utilizing advancements in language models to model the sequence–structure relationship. The remarkable precision of AlphaFold^20,21^ and the availability of its source code have revolutionized computational and structural biology. Although initially designed for monomeric structures, AlphaFold intrinsically demonstrated a notable capacity for predicting protein complexes through input manipulation, such as adding a linker to the protein. While these input-adapted versions outperform previous state-of-the-art methods, the recently introduced retrained AlphaFold-Multimer system further improves interface predictions to 58% in a recent benchmark.^22^ However, AlphaFold is limited in its ability to predict structures for polypeptides that do not conform to the one-sequence/one-fold rule, especially relevant for disordered proteins. The implications of IDP prediction using DL tools have been reviewed.^23,24^ More understanding is needed on the relationship between the confidence metrics generated by the DL tools and the dynamic behavior of the predicted structures. Recent research has shown that the predicted alignment error^21^ is correlated to the dynamics of the protein in MD simulations and the root-mean-square fluctuation (RMSF) is related to the predicted local distance difference test.^25,26^ Another recent preprint showed that the inter-residue distances predicted by AlphaFold for disordered proteins can be used as a prior to construct accurate structural ensembles with MD simulations.^27^

External (oscillating) electric fields can be employed in MD simulations to study their effect on biomolecules.^28^ Todorova et al. found that electric fields have a strength-dependent influence on the secondary structure and dynamics of amyloidogenic peptides.^29^ Further studies investigated the effects of varying frequencies on aggregation propensity, with a 1 GHz field at a low strength (around 10 mV/nm) trapping the peptide in a specific conformation. Meanwhile, higher strengths, of 700 mV/nm but already at 70 mV/nm, showed a breakup of the hairpin conformation.^30^ Simulations of the Aβ40 peptide under a static electric field showed a transition from α-helical to β-stranded structure.^31^ Short Aβ42 fibrils (pentamers) showed a partial degradation of the first β-strand segment due to the disruption of their charged N-termini.^32^ A thorough review on the simulations of biomolecules under electric fields has been published recently.^33^

A very recent study has investigated the effects of oriented external electric fields on the aggregation of oligomers of a 7-residue segment of the β-amyloid peptide.^34^ The authors used the Aβ42 heptapeptide segment K_16_LVFFAE_22_ which aggregates into plaques faster than the full sequence. Ten peptides in a simulation box were allowed to aggregate for 500–1000 ns. Afterward, both static and oscillating electric fields were applied to study the degradation of the peptide aggregation. The oscillating field was applied at a high strength of 200 mV/nm, with frequencies of both 0.1 and 1 GHz. In both cases, a thorough disaggregation was observed. Upon removal of the electric field, the peptides did not aggregate back. The authors therefore concluded that microwave radiation can revert amyloid aggregation in a nonreversible manner.

Here, we set out to study the stability of the minimal β-amyloid toxic species in the presence of an external oscillating electric field in the middle of the microwave range. We first apply deep learning (DL) tools (AlphaFold-Multimer and ColabFold) to predict model structures of the Aβ42 dimer which is the smallest toxic oligomer. We then use MD simulations to analyze the flexibility of the predicted structures. The top models are structurally stable in 50-ns MD runs and compare to highly populated clusters of μs scale MD simulations (published by others^15^). Next, we use the highest-confidence prediction to test the effect of an oscillating electric field on the behavior of the Aβ peptide dimer. The 100−300 ns simulations reveal a field-strength-dependent decay of the β-sheet content. Control simulations using the HY5 leucine zipper of *Arabidopsis thaliana* show a slower degradation and only upon application of the strongest external electric field.

## 2 Results and Discussion

We predicted the structure of the Aβ42 dimer using two deep learning tools, AlphaFold-Multimer and ColabFold. We first analyze the 50-ns runs (abbreviated as ns-MD in the following) for quantifying the flexibility of the structures predicted by deep learning. We then compare the predicted structures with the publicly available microsecond sampling^15^ which we call μs-MD in the following. Finally, we analyze the kinetics of secondary structure decay under the influence of an external electric field (EF-MD simulations of 100−300 ns).

### 2.1 Prediction of Dimeric Aβ42 by Deep Learning

**Figure 1:**
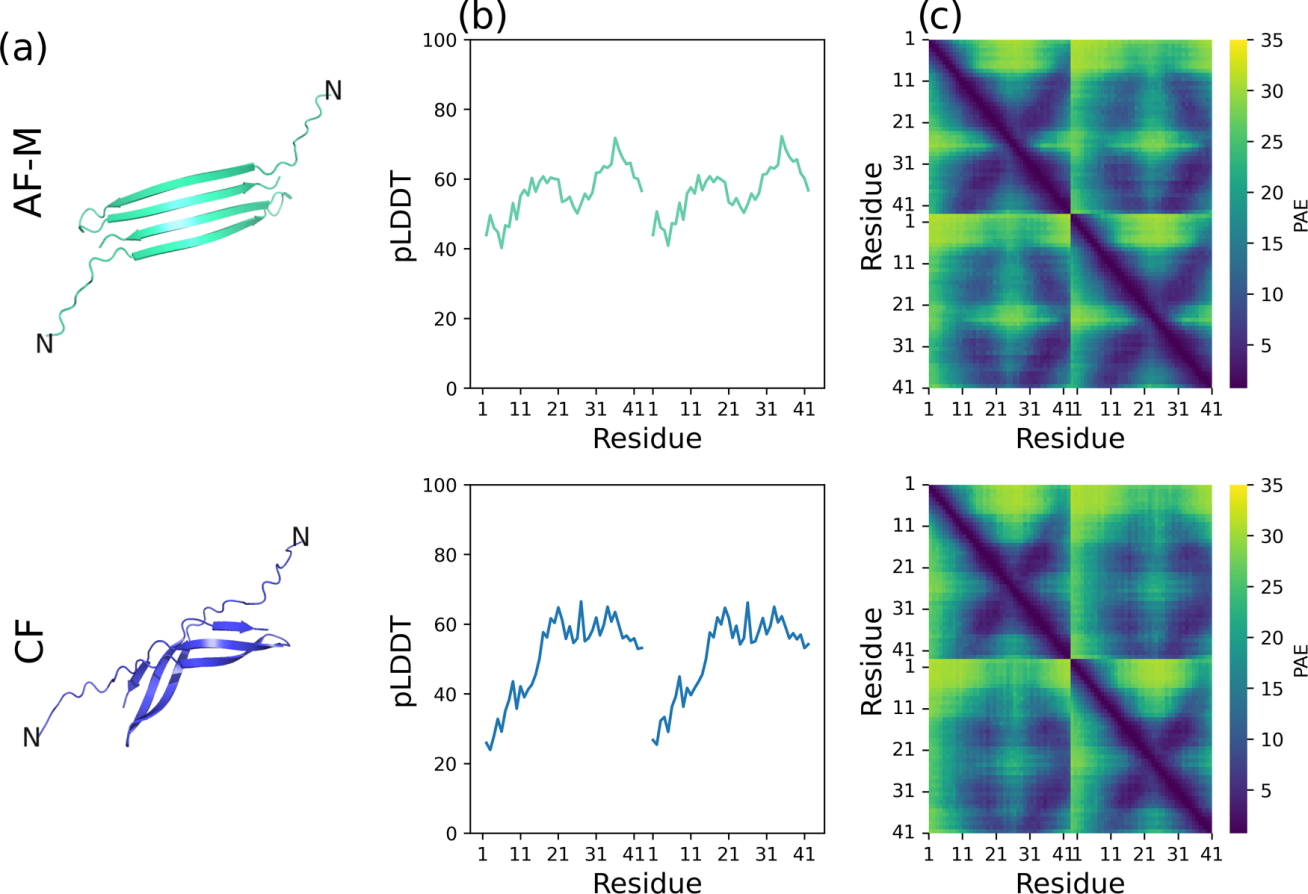
(a) Model structures of the Aβ42 dimer predicted by AlphaFold-Multimer (AF-M) with 3 recycling steps and ColabFold (CF) with 50 recycling steps. (b) Predicted local distance difference test (pLDDT)^26^ score for the C_α_ atoms. A higher value indicates higher confidence. (c) Matrices of predicted aligned error (PAE). In each matrix, the intrachain confidence is shown in the top-left and bottom-right quadrants while the interchain, i.e., dimeric interface, confidence is shown in the top-right and bottom-left quadrants. Low PAE values indicate high confidence.

The top-ranking AlphaFold-Multimer (AF-M) and ColabFold (CF) models were chosen for further analysis (Figure 1). Both structures have a similar fold, although the AF-M is more planar, while the CF prediction resembles part of a β barrel (Figure 1a). The overall confidence, as measured by the predicted local distance difference test (pLDDT) score,^26^ is relatively low for both predicted structures (Figure 1b). The low scores are below the pLDDT threshold of 70, which is reported by the authors of the DL tool as threshold for a “generally correct” backbone prediction.^20^ This is not surprising given the Aβ42 peptide has a disordered nature, something for which the DL tool is not trained. The score is stated to estimate the local agreement to an experimentally solved structure; therefore, a low score can be seen as a conformationally “diverse” region of the prediction. The low score is congruent with the description of Aβ42 as an ensemble of structures. Furthermore, the pLDDT depends on the information given by the depth of the multiple sequence alignment (MSA) used to find coevolutionary information on the sequence.^35^ Only 81 homologous sequences were found by MMseq2 and for the MSA of Aβ42 and 131 unique sequences with jackhammer. This means the MSA is shallow in both cases, which can lower the confidence of the model.^21^ Both predictions yielded β hairpins as observed in previous simulation studies.^12^ The two β hairpins form an antiparallel 4-stranded β-sheet in both the AF-M and CF models. The AF-M prediction has a slightly higher confidence overall. This is probably because the strands are continuous on the AF-M structure, while they are interrupted in the middle in the CF model. These arrangements are similar, but not identical, to a ″dimeric base″ arrangement which has been proposed as the only seed for toxic Aβ42 oligomers.^17^ Interestingly, the 7-residue stretch K_16_LVFFAE_22_ of the Aβ42 peptide which was studied by Kalita et al. is predicted as β strand in both structures.

The predicted aligned error (PAE) is a matrix whose elements reflect the confidence of the model at the level of individual pairs of residues. It illustrates the model confidence in the contacts inside a domain (diagonal quadrants of the plot) and in the interaction between domains (off-diagonal quadrants).^22^ The confidence on the intrachain structure is slightly higher than the dimeric interface (Figure 1c). The AF-M and CF model structures show similar confidence for the β-strand regions of each chain and for the interchain contacts. The PAE for the N terminal segments of both chains is higher with respect to other residues, meaning their localization is of low confidence, i.e., random. This can be explained by their large flexibility.

**Figure 2:**
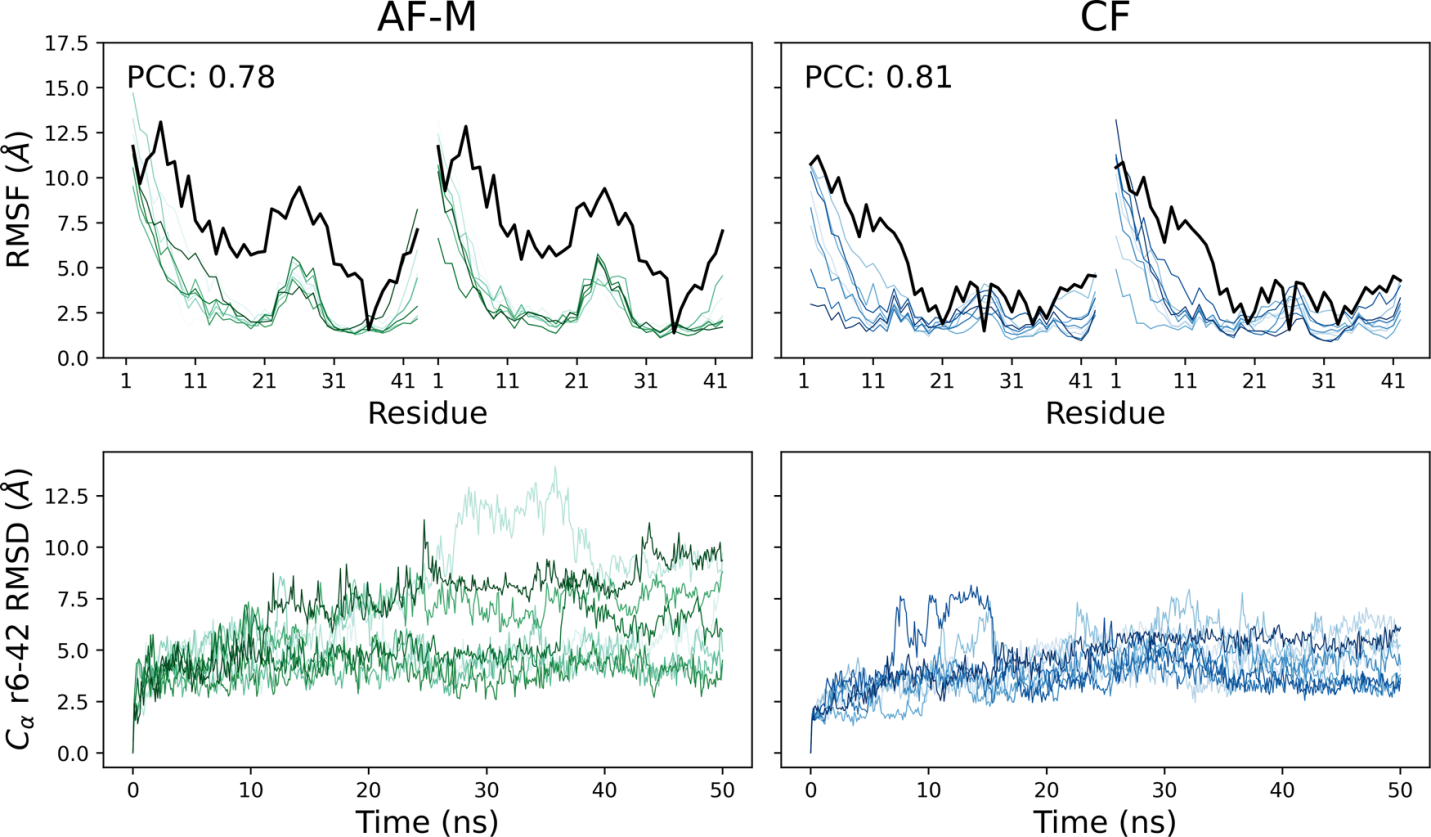
Dynamic behavior of the AlphaFold-Multimer model (AF-M) and the ColabFold (CF) model. (Top) Sequence profile of the RMSF of the nonhydrogen atoms along the eight ns-MD runs (green, AF-M; blue, CF). The reverse pLDDT normalized to the RMSF is also shown (black). The Pearson correlation coefficient (PCC) is shown as inset. (Bottom) Time series of RMSD from the model structure. Residues 1–5 were ignored as they show large fluctuations for both models.

The dynamic behavior was explored through the root-mean-square fluctuation (RMSF) and root-mean-square deviation (RMSD) of the ns-MD simulations started from each model (Figure 2). Most of the fluctuations are seen in the N terminal segment, so the RMSD was calculated only on residues 6 to 42 to avoid excessive noise. The CF model shows a lower RMSD than the AF-M one in the eight ns-MD trajectories, denoting higher stability of the predicted conformation. Consistently, the CF model structure has lower fluctuations along the ns-MD. The higher rigidity might originate from the β-barrel-like structure, which keeps the flexible loops in place more firmly than the flat arrangement of the AF-M model. The predicted confidence (pLDDT) anticorrelates with the backbone flexibility as shown by the sequence profiles of the renormalized pLDDT (i.e., reverse normalized pLDDT, see the Methods section) and the RMSF (Figure 2, top). A similar anticorrelation in the sequence profiles of the pLDDT and RMSF has been reported for globular proteins.^25^

**Figure 3:**
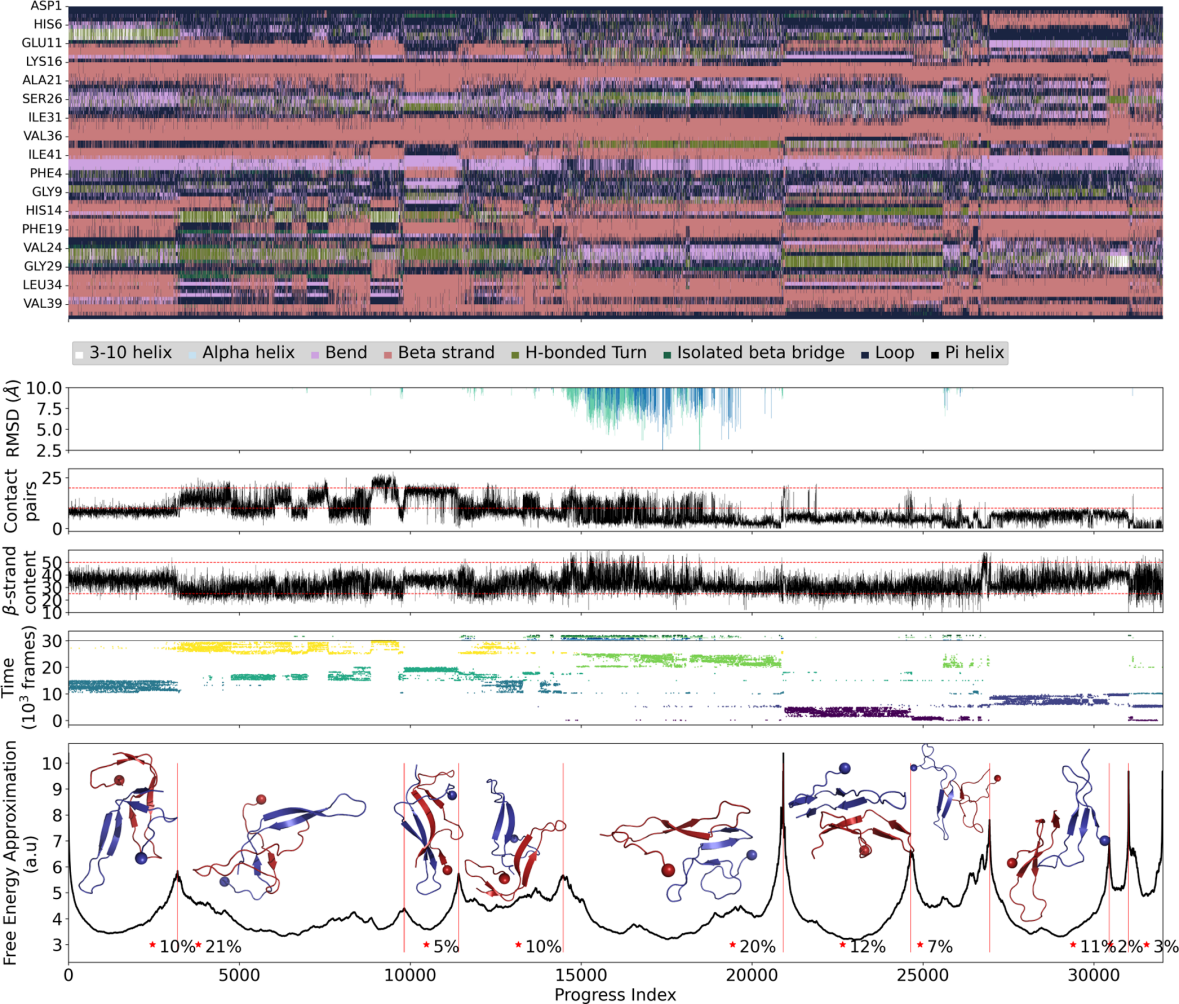
SAPPHIRE plot of the μs-MD (6) sampling of the Aβ42 dimer^15^ and the ns-MD simulations of the predicted models. (Top) Geometric annotation: Sequence profile of secondary structure. (Middle) Geometric annotations: C_α_ RMSD from the CF (blue) and the AF-M (green) structures; number of interchain contacts with a distance lower than 5 Å (black line, red reference lines at 25 and 50 contacts, respectively); number of β-strand residues (black line, red reference lines at 10 and 20 residues). (Bottom) Temporal annotation (dots) illustrates the position of each frame of the trajectory along the progress index. The colors of the dots reflect the six independent runs from the μs-MD and the 16 runs of the ns-MD which are separated from the μs-MD by a thin black line. The cut-based free energy profile illustrates the transitions between states (black).^36^ The insets show the centroid structure from each basin, the N terminal residue is marked by a sphere. The position of the centroids along the progress index is marked by a red star. The percentages show the relative weight of each free energy basin. Basins are delimited by red vertical lines.

To further validate the DL predictions we compared them to the equilibrium sampling of Aβ42 dimers reported by others. Fatafta et al. have generated a total sampling of 6 μs of the Aβ42 dimer in explicit solvent, and these data are publicly available. We refer to this sampling as μs-MD. We use the SAPPHIRE analysis to compare the DL-predicted structures and the μs-MD sampling (Figure 3). The SAPPHIRE analysis is an automatic and unsupervised tool for determining the free energy basins of a complex system and quantifying the number of transitions between them.^36,37^ It also provides a fully data-driven clustering of the phase space.^38^ Briefly, the SAPPHIRE analysis consists of a reordering of the trajectory snapshots based on geometric similarity (called progress index) with the assumption that structural similarity corresponds to kinetic proximity. Here, we employ interchain C_α_ distances for the reordering of the snapshots. The cut function (black solid line in the bottom part of Figure 3) is an approximation to the free energy profile, which is very useful for identifying the barriers between basins.

The central basin (20% weight, progress index value between 14,500 and 21,000) is populated mainly by the fifth μs-MD run and is visited also by the other μs-MD runs. Importantly, the ns-MD sampling (started from the AF-M model) is almost fully included in the central basin which consists mainly of conformers with antiparallel arrangements of the interchain contacts. The remaining free energy basins include conformations with a substantial amount of β-stranded secondary structure but, unlike the DL models, the interchain contacts have a parallel arrangement.

The geometric annotation shows that the snapshots on the right of the central basin have generally less than 10 contact pairs between the chains, while to the left, the number of contacts oscillates between 10 and 20. The content of β-strand residues is slightly larger in the central basin than in the remaining sampling. The small basin on the extreme right, with progress index around 32000, corresponds to the unbound chains, as shown by the lower number of contact pairs. The C_α_ RMSD of the DL models from the μs-MD simulation snapshots ranges between 5 and 10 Å in the central basin, which together with the location of the ns-MD frames in this basin, show that the DL predictions resemble states visited by the μs-MD simulations.

As each of the two DL models has an identical structure for the two chains, we also used the SAPPHIRE analysis and its associated plot to compare the DL models and the μs-MD sampling at the single-chain level. For this, we extracted the coordinates of each chain separately and then concatenated them, treating the concatenated sampling as a single 12 μs trajectory of an Aβ42 monomer (Figure S1). The two DL models are similar to the representatives of the most populated free energy basin of the μs-MD sampling which are characterized by a β-hairpin conformation. These results are consistent with the analysis at the dimer level (Figure 3). Thus, the comparative analysis of the DL-predicted structures with the ns-MD and μs-MD simulations provides evidence that both DL structures are good candidates as starting points for MD simulations in the presence of an external electric field.

Finally, we sought to validate our ns-MD simulations with experimental data. Circular dichroism (CD) spectra were predicted^39^ for the frames of the AF-M ns-MD and μs-MD from several basins of the SAPPHIRE plot (Figures S2 and S3). They were then compared to experimental CD spectra from the literature (Figure S4). A caveat for this comparison is that the predicted spectra are generated for individual structures, while CD measures the ensemble of protein in the sample being analyzed. The CD spectra predicted from the ns-MD runs suggest a mixture of disordered loops and β-strand content which is consistent with the experimental spectra acquired for Aβ42 at concentrations of 25^40,41^ and 50 μM^42^ at early time points. This corresponds to the oligomeric state of the amyloids at the beginning of the measurements, before they start aggregating into fibrils. Therefore, the predicted ns-MD CD spectra help validate these conformations as similar to those of experimental Aβ42 oligomers.

### 2.2 Effect of Electric Field on Aβ42

The top structure predicted by AF-M was chosen as starting conformation because it presented a higher confidence (pLDDT) than the best CF model. The residues 1–5 were neglected as they show very high fluctuations (Figure 2) and they are disordered in the fibrillar structures of Aβ42 determined by solid-state NMR spectroscopy.^43,44^ A total of 16 independent MD runs, which we call EF-MD, were started under the influence of an oscillating electric field of different strengths (0, 10, 100, and 200 mV/nm) with a frequency of 1 GHz. Each of the 16 copies of the four systems was initially simulated for 100 ns. Some systems were extended to better differentiate the behavior between different simulation parameters.

**Figure 4:**
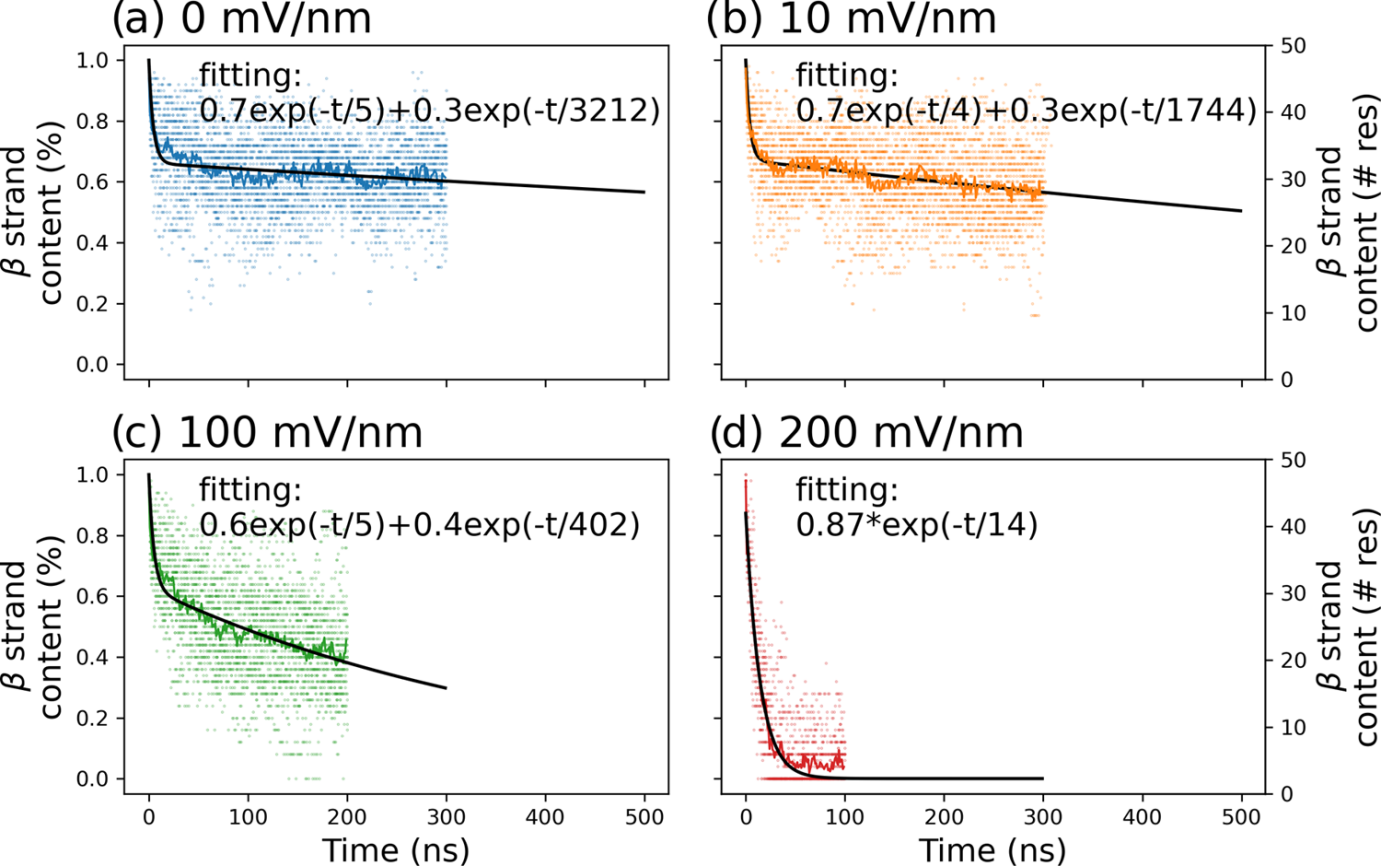
Decay of the β-strand content. (a–c) Two-exponential function (black) was fitted to the average β-strand secondary structure content (colored lines). The individual values for each of the 16 trajectories are also shown (colored dots). (d) At the highest field strength, a single-exponential function can be employed to fit the decay.

We focus the analysis on the β-strand content as the fibrillar structures of Aβ consist of β sheets.^45,46^ Moreover, α-helical structure was observed in the EF-MD simulations only transiently for short periods of time (Figures S5–S8). There is a significant decay of the β-strand content in all simulations, and the rate of decay correlates with the field strength (Figure 4). Furthermore, after an initial fast decay, the rate of the slower phase depends on the field strength. The initial fast decay takes place in the first 5 to 10 ns of simulation, where the β-strand content drops from 50 to around 35 residues. The β-strand content in the initial phase of decay is similar to the one observed in the central free energy basin of the SAPPHIRE plot (Figure 3). An analysis of the number of intra- and intermolecular hydrogen bonds in the dimer suggests a possible explanation for the disruption of the secondary structure caused by the electric field (Figures S5–S8). The polar groups of the solute, e.g., backbone NH and CO, have a fixed dipole moment in the classical (i.e., nonpolarizable) force field. Thus, they can respond to the change in the external electric field only by reorienting themselves.^47^ The Aβ(6–42) peptide dimer cannot rapidly rotate to adjust to the changing direction of the field, while the water molecules can rotate rapidly in the sub-nanosecond time scale. As the strength of the field increases, the hydrogen bonds between β-strands break and there is an increase in the number of peptide–water hydrogen bonds.

**Figure 5:**
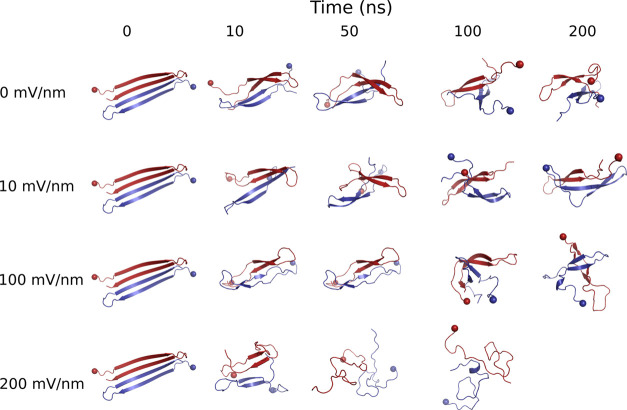
Snapshots from single EF-MD simulations showing the decay of secondary structure along time (*x*-axis) for different strengths of the external electric field (*y*-axis). The two Aβ42 peptides are shown by different colors (blue and red). The N-termini are marked by spheres. The fast degradation is exemplified by the snapshot of the 200 mV/nm EF-MD trajectory at 50 ns, where no β-strands are visible.

Figure 5 shows representative snapshots from the EF-MD simulations at different strengths of the electric field. The cartoon representation helps to visualize the rapid degradation of secondary structure and the correlation between rate of decay and field strength (see also Supporting Information, Movies S1–S4). There is a gradual deterioration of the intrachain and interchain β-strand structure at all field strengths. Short, mainly one-turn, α-helical segments are observed sporadically (Figure 5). Furthermore, there is a monotonous decrease of symmetry from the initial AF-M model structure in which the two peptide chains have identical structures.

The decay in the mean β-strand content can be fitted by a two-phase model up to a field strength of 100 mV/nm (black line Figure 4). As mentioned above, there is a fast loss of secondary structure to a level of around 70% (35 residues), followed by a slower decay. The fast decay channel reflects the relaxation of the initial structure. The slow channel captures the voltage-dependent degradation of β-strand content. For the EF-MD with no external field, the mean lifetime is very large at around 3 μs. A weak field of 10 mV/nm results in a shorter lifetime of 1.7 μs while a field of 100 mV/nm shortens the lifetime to about 0.4 μs. A large variability is observed for the 16 independent EF-MD runs at each of the field strength values. At field strengths of 0 and 10 mV/nm the β-strand content ranges between 40 and 80% after 0.3 μs. At a field strength of 100 mV/nm, there is a 20–60% range of β-strand content after 0.2 μs. In contrast, at the highest field strength (200 mV/nm), the extremely fast decay can be modeled by a single exponential and a lifetime of 0.014 μs. The preexponential factor is close to one which supports the choice of the single-exponential model at the field strength of 200 mV/nm. Overall, the simulation results provide evidence for a substantial loss of β-strand content in dimeric Aβ42 within a 1 μs time scale for 1 GHz alternating electric field of strength higher than 10 mV/nm.

### 2.3 Control Simulations with a Helical Dimer

**Figure 6:**
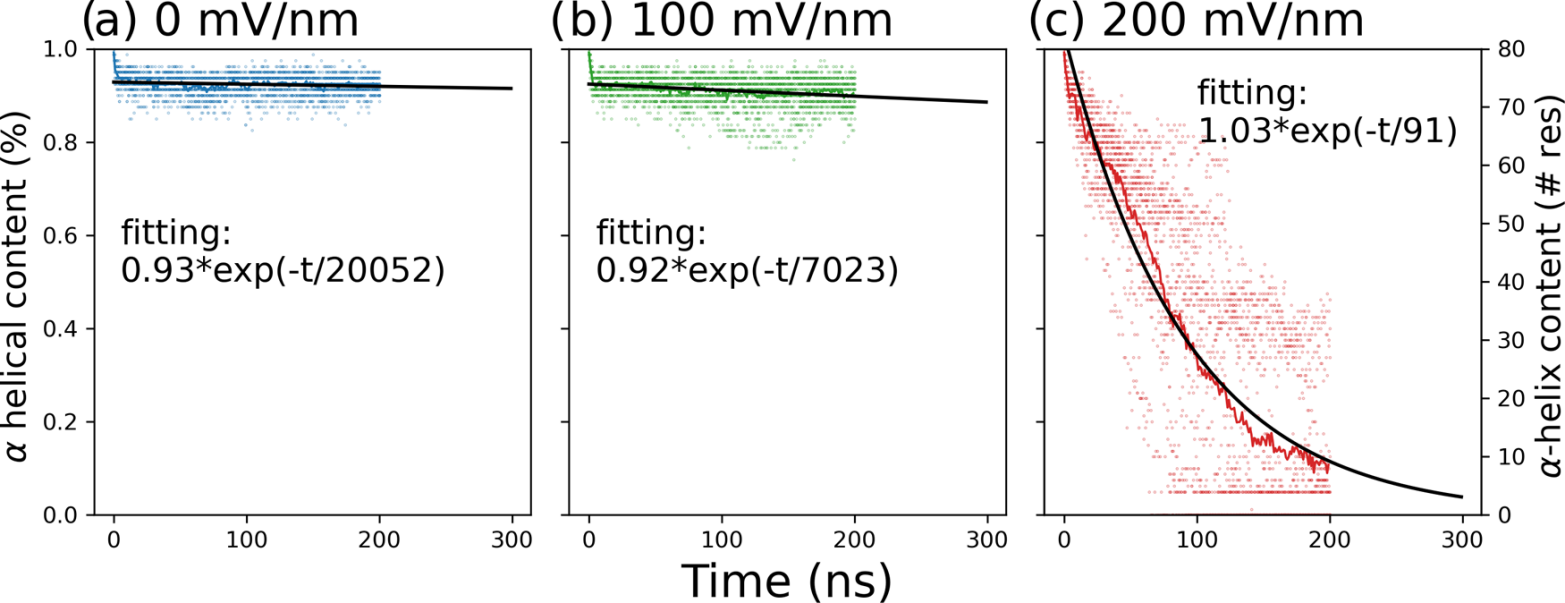
Slow decay of the α-helical content of a leucine zipper. (a, b) No degradation is observed at the simulations without and with an external electric field of 100 mV/nm. (c) Degradation at the 200 mV/nm field strength.

We then asked the question if a dimeric system with a different secondary structure shows similar kinetics of structural disruption. To answer this question we have simulated the HY5 leucine zipper from a transcription factor of *A. thaliana* (PDB ID: 2OQQ) which has an α-helical dimeric topology. This peptide segment was chosen as it has the same number of residues of the (nontruncated) Aβ42. External electric fields at 100 or 200 mV/nm and an oscillation frequency of 1 GHz were employed in these control simulations. The leucine zipper showed no degradation at a field strength of 100 mV/nm during the 200 ns of simulation for any of the independent runs. At 200 mV/nm, degradation of the α-helices is observed but it is significantly slower than for the Aβ dimer (Figure 6). The number of intra- and intermolecular hydrogen bonds does not decrease in the simulations at 0 and 100 mV/nm electric fields (Figures S9 and S10). At the field strength of 200 mV/nm, there is a replacement of the intrasolute hydrogen bonds with solute–solvent hydrogen bonds (Figure S11). It is important to note that the leucine zipper is a folded peptide which is stable enough to crystallize, while Aβ42 is an intrinsically disordered peptide. Further studies using peptides and proteins of different topologies, disordered/globular, mixed β-sheet, and α-helix, could shed light on the behavior of different proteins under external electric fields.

## 3 Conclusions

There is substantial experimental evidence for the toxic role of the early oligomers in amyloid-like diseases.^4,5^ The smallest toxic species of the Alzheimer’s disease-related Aβ peptide is the dimer.^7^ β-Sheet containing Aβ42 has been shown to be essential for neurotoxic oligomer aggregation. Disrupting the β-strand content in Aβ42 oligomers is of clinical interest.^9,17^ A large number of antibodies and small molecule inhibitors of Aβ oligomerization and/or amyloid fibril formation have failed in clinical trials in the past decade. Can electric fields be applied to break down toxic aggregates? Are MD simulations adequate for investigating the stability of the early oligomers in the presence of an oscillating (or static) electric or electromagnetic field?

As MD simulations require starting conformation(s), one challenge is that monomeric Aβ is an IDP and very little is known on the conformations populated by the dimer. Recent advances in deep learning have allowed the rapid generation of structural models for globular proteins.^20,21^ These predictions must be taken carefully particularly for IDPs, as the DL neural networks have been trained on structured proteins.^23,24^ In the present study, the power of DL tools was exploited to generate predictions for the structure of Aβ42 dimers. We have first compared the predicted structures of the dimers against previous μs-MD simulations. The DL predictions are congruent with the sampling obtained by μs-MD simulations as they are close to highly populated conformations of the dimer. Validation with the ns-MD simulations shows that both predicted structures of the Aβ42 dimer have a high structural stability particularly at the segment 6–42. Comparison to experimental results reveals a secondary structure content that is similar to that observed in ensemble measurements by circular dichroism. These findings give us confidence on the use of a DL-generated model as the initial structure for our EF-MD simulations.

Then, to try to answer the questions posed above, we have performed MD simulations in the presence of an external oscillating field using the DL model with the highest prediction confidence as starting structure. We have focused the analysis of the MD trajectories on the effect of electric fields of different strengths on the secondary structure content of the homodimer. The simulations provide evidence for a direct relationship between the strength of the field and the rate of decay of the β-strand content.

Previous studies have shown that the application of an external electric field would prevent the formation of hairpin structure of the apoC-II (60–70) peptide.^30^ Furthermore, in a simulation study of oligomers of the heptapeptide segment Aβ(16–22) degradation of the secondary structure content was observed upon application of oscillating electric fields (of strengths 100 mV/nm at 0.1 GHz and 200 mV/nm at 1 GHz).^34^ There are major differences between the work by Kalita et al.^34^ and our study. First, they simulated the 7-residue segment Aβ(16–22) while we investigate the segment 6–42 of Aβ (residues 1–5 are disordered in several fibrillar structures). Shorter zwitterionic peptides have a higher susceptibility to the external electric field than a longer peptidic chain which is also more similar to the biological species. Second, we focused on the minimal toxic species, i.e., the dimer, while the previous study used 10 copies of Aβ(16–22) which can form a protofilament. Third, we used a starting conformation of the dimer generated by DL while Kalita et al. started from random positions and orientations of the peptides. Fourth, Kalita et al.^34^ investigated mainly the geometric properties of the heptapeptides while we focus on the kinetics of the β-content decay.

Our simulation results for the minimal toxic species, i.e., the Aβ(6–42) dimer, provide evidence that the application of an oscillating (at 1 GHz) external electric field of 100 mV/nm (or higher) results in the rupture of β-sheet content in a μs time scale. A significantly slower decay of β-sheet content is observed at a field strength of 10 mV/nm. Thus, the wide range of electric field strengths used in the present study provides a nuanced picture of the effect of the external field on the β-sheet structure of the Aβ homodimer. As a control, we have simulated the helical dimer of a leucine zipper which shows substantially higher structural stability than the Aβ homodimer as decay of the zipper helical content is observed only at the field strength of 200 mV/nm. Individual water molecules can rotate faster than the Aβ homodimer or leucine zipper to optimally align with the external electric field. Thus, degradation of the regular elements of secondary structure is promoted by a loss of intrasolute hydrogen bonds and an increase in solute–solvent hydrogen bonds.

Because of the computational cost of μs MD sampling, we have chosen only one structure as starting point for the MD simulations in the presence of the electric field. This could be a potential limitation of our study given the disordered nature of the amyloids. A diverse set of initial structures could be considered for further simulation studies. Furthermore, the effect of electric fields could be studied on globular proteins consisting mainly of β-sheets or α-helices. Very little is known on the reversibility of the folding of globular proteins under oscillating fields. Since the time scale of folding of most globular proteins is in the millisecond range, it is not possible to investigate the influence of external electric fields on (un)folding with conventional atomistic simulations. In future simulation studies, it will be of interest to analyze the effect of the external oscillating electric fields on the structure of (oligomeric) Aβ42 at the membrane. Fatafta et al. have sampled the conformational space of dimeric Aβ42 at a lipid bilayer that reflects the composition of neuronal membranes.^15^ This sampling could be used as starting point for a simulation study in the presence of an electric field. Due to the low dielectric constant of lipid bilayers (around 3, ref (48)) compared to that of water at physiological temperature (78.5), one can expect a stronger influence on the electrostatic interactions between polar groups of Aβ42.

Another important caveat is that the loss of β-sheet content in the (early) oligomers of Aβ might not be necessarily beneficial. Stabilization of the cross-β fibrils of the prion protein by small molecules has shown a therapeutic effect in mice models of the prion disease.^49^ Thus, the rupture of β-sheet content might even be counter-productive as it might promote fragmentation which could result in a larger number of toxic oligomers. The complexity of the Aβ self-assembly process (e.g., presence of kinetic traps and kinetic control of amyloid fibril formation) and the very small knowledge of the toxic species are major challenges in the development of therapeutic agents for Alzheimer’s disease.^50^

In conclusion, the use of oscillating electric fields is a promising new avenue of research into the degradation of amyloid oligomers. The radiofrequency chosen in this study (1 GHz) is comparable to that observed by everyday appliances like smartphones, although at a higher field strength. The present simulation results should spur further characterization of external electric fields on (neuronal) cell lines, brain organoids, and animal models. Experimental evidence for a direct link between reduction of symptoms (e.g., improved memory in rodents) and electric field treatment could open the path to a novel therapy for Alzheimer’s disease.

## 4 Methods

### 4.1 Modeling

The AlphaFold-Multimer deep learning tool was used to predict the structure of homodimeric Aβ42 (two chains of D_1_AEFRHDSGYEVHHQKLVFFAEDVGSNKGAIIGLMVGGVVIA_42_). Two independent predictions were carried out. The first prediction was generated using a local installation of the AlphaFold-Multimer (AF-M) v2.1 with default settings in which the multiple sequence alignment (MSA) was performed on a reduced version of the BFD database optimized for speed and hardware requirements.^21^ Due to the disordered nature and the high flexibility of the Aβ42 peptide, a second modeling session with a high number of recycling steps was performed. Recycling executes the network multiple times by re-embedding the 3D structure to the pairwise distances representation as input.^21^ A higher number of recycles has been reported to increase the quality of models of interacting proteins.^51^ The second prediciton was generated with ColabFold (CF). CF is an implementation of AlphaFold that can be run on Google Colaboratory^52^ without downloading the databases for generating multiple sequence alignments. Instead, fast and sensitive MSAs are created with MMseqs2 by searching homologous sequences in the uniref30 and environmental database (ColabFoldDB).^53^ A total of 50 recycling rounds were carried out for the CF prediction. No template was used for modeling. The prediction with the highest model confidence (0.8 pTM + 0.2 ipTM) was chosen for further validation.

### 4.2 Validation of Models

Despite being fast and easy to use, the validity of computationally predicted models can be put to question. Therefore, the DL-generated models have been validated against the extensive 6 μs MD sampling reported previously by Fatafta, et. al.,^15^ which we refer to as μs-MD. The trajectories were obtained from https://data.mendeley.com/datasets/92mkp4pk86. A SAPPHIRE analysis^37^ was performed to assess the similarity of the monomer of the DL models and the μs-MD sampling (Figure S1). As the two peptides have identical structures in the AF-M model, only the first peptide in the file of the predicted coordinates was used, and similarly for the CF model. Concerning the μs-MD, the coordinates of the individual chains of the homodimer were extracted, and the trajectory was concatenated. The resulting single-chain trajectory, consisting of a total sampling of 12 μs, was then analyzed using SAPPHIRE-based clustering.^38^ Distances between C_α_ atoms of the peptide were chosen to build the progress index. Per-frame locally adapted weighted distances were used.^54^

The dynamic stability of the obtained models was checked by molecular dynamics simulations. For each predicted structure, eight independent 50-ns MD simulations were started. We call these ns-MD simulations. All ns-MD simulations were performed with GROMACS 2021.5 using the CHARMM36m July 2021 force field. The models were solvated and equilibrated with Na+ and Cl- ions to a concentration of 150 mM. Energy minimization was applied, and a canonical equilibration under positional restraints was performed for 5 ns to reach 300 K. Each system was simulated for 50 ns to study the behavior of the homodimer. The stability of the predicted structure was evaluated by common metrics, namely, the C_α_ RMSD to the initial frame after equilibration using residues 6–42 of each chain, and the heavy-atom RMSF against a sliding-average structure calculated every 5 ns. Furthermore, the RMSF was correlated (Pearson correlation coefficient) to the reverse normalized (renormalized) pLDDT confidence metric generated with the prediction, by subtracting the maximum value and dividing by the range, as has been previously reported.^25^ We predicted circular dichroism (CD) spectra for the AF-M ns-MD frames and some μs-MD frames using PDBMD2CD.^39^ The spectra calculated by using as input the MD snapshots were compared to the experimental CD spectra.

A second SAPPHIRE analysis was then performed on the dimeric ensemble (Figure 3). A subsampling of 200 ps was used for the ns-MD trajectories, as it is the time step of the μs-MD trajectories. Interchain C_α_ distances were calculated for the 30,000 μs-MD frames and the 2000 ns-MD frames individually and then concatenated to avoid errors due to differences in simulation box sizes and shape. The time series of distances was given as input features to CAMPARI’s NetCDF miner. A sigmoidal distance transformation was applied to reduce the influence of high euclidean distances between the frames, centered on 40 with a slope of 20. Principal Component Analysis was then applied, and the two main principal components were kept for the construction of the progress index. All SAPPHIRE analyses were performed using CAMPARI (V4).^55^ A secondary structure prediction (dssp) was used as geometric annotation for both SAPPHIRE plots. The RMSD of the AF-M and CF structures against the trajectories was calculated, considering C_α_ atoms only, to find to which basin of the SAPPHIRE plot the predicted structure would belong. Centroid structures were calculated, defined as the structure of the frame with the lowest euclidean distance to the mean of each basin.

### 4.3 Effects of Electric Field on Aβ42

#### 4.3.1 Simulation Setup

Multiple MD runs under the influence of an external oscillating electric field (EF-MD) were started from the top-scoring model of the Aβ42 dimer obtained with the AlphaFold-Multimer (AF-M). All simulations were performed with GROMACS 2021.5^56^ using the CHARMM36m force field.^19^ The two chains were shortened by removing the initial five residues, therefore starting on His_6_. This is due to the high flexibility of these residues as shown from the initial validation (Figure 2). The model was solvated in an 8.1 nm cubic box of water molecules, and Na^+^ and Cl^–^ ions at a concentration of 150 mM. Afterward, the system was subjected to energy minimization. A 5 ns NVT equilibration was performed, in which the system was kept under positional restraints, to reach 300 K. For the production MD, four conditions were evaluated, with the aim to test the effect of an oscillating electric field on the secondary structure of the dimer. The behavior with no electric field was compared to that after subjecting the system to oscillating fields of 10, 100, and 200 mV/nm. All fields were applied with a frequency of 1 GHz, i.e., the direction of the field was rotated by 180° every ns. The field was applied from one of three directions, on the x, y, or z plane. The field was applied by setting the respective electric field-(*x*/*y*/*z*) field in the GROMACS input file. Sixteen independent simulations were started for each of the systems applying the electric field in one plane. The direction of the field was assigned to each copy of the system in a sequential manner. The simulations were run initially for 100 ns. The systems subject to 100 mV/nm electric field were extended to 200 ns, while the 0 and 10 mV/nm were extended to 300 ns. The same setup was used to test the stability of the leucine zipper α-helical homodimer of the HY5 transcription factor of *A. thaliana*. The peptide monomer has a length of 42 residues. The crystal structure of the leucine zipper homodimer has a resolution of 2.0 Å (PDB code: 2OQQ) and was used as starting structure for the MD simulations. Production runs were carried out for 200 ns and electric fields of 100 and 200 mV/nm were employed at the same frequency as for the Aβ42 homodimer (1 GHz).

#### 4.3.2 Analysis

The effect of the electric field was studied by monitoring the presence of the β-strand secondary structure elements in the dimer. For each EF-MD trajectory, the β-strand content was predicted using the *mdtraj* package’s *dssp* function.^57,58^ The time series of the strand content was calculated for each trajectory and averaged per system. A two-exponential model was used to fit the time-dependent decay of the mean β-strand content across the eight simulations for each potential. The extremely rapid decay at 200 mV/nm can be fitted by a single exponential. For the leucine zipper, the α-helical content was monitored and a single-exponential function was fitted to the time series of the average number of helical residues. Hydrogen bond content was quantified using the Wernet–Nilsson algorithm implemented in *mdtraj*.^58,59^

## Supporting Information Available

Validation of structures: monomeric SAPPHIRE analysis, circular dichroism analysis; effect of electric fields: secondary structure of EF-MD and leucine zipper (PDF)
Trajectory with no electric field (Movie S1) (MP4)
Trajectory with electric field of 10 mV/nm strength and 1 GHz frequency (Movie S2) (MP4)
Same as Movie S2 with a strength of 100 mV/nm (Movie S3) (MP4)
Same as Movie S2 with a strength of 200 mV/nm (Movie S4) (MP4)
