# Supplementary material for: Disrupting Dimeric β-Amyloid by Electric Fields
Source: ACS Phys Chem Au. 2023 Jul 12;3(5):456–66. doi: 10.1021/acsphyschemau.3c00021 (PMC10540290; doi:10.1021/acsphyschemau.3c00021)
Supplement: Supplementary file 1 — pg3c00021_si_001.pdf [file pg3c00021_si_001.pdf]

Supporting Information: Disrupting dimeric β-amyloid by electric fields

Pablo Andrés Vargas-Rosales, Alessio D'Addio, Yang Zhang, and Amedeo Caflisch*

Department of Biochemistry, University of Zurich, CH-8057 Zürich, Switzerland

E-mail: caflisch@bioc.uzh.ch

Phone: +41 44 635 5521

Validation of deep learning (AlphaFold) models

Comparison to MD simulations of dimeric Aβ42. A preliminary SAPPHIRE analysis was performed on the single chains to assess the convergence of the μs-MD trajectories. For this purpose, the coordinates of each of the two Aβ42 chains were extracted, and concatenated to generate a trajectory of a single chain of cumulative 12 μs. Intra-chain Cα distances were used to build the progress index. From the SAPPHIRE analysis, we observe that both AF-M and CF model structures belong to the most populated free-energy basin of the μs-MD sampling (Figure S 1). The CF model is within 5 Å RMSD of 232 frames (0.38% of total sampling) while the AF-M model is within 5 Å RMSD of 90 frames (0.15% of total sampling). A threshold of 10 Å yields a 25% and 20% of the total μs-MD sampling similar to the CF and AF-M structures, respectively. The majority of these frames where the predictions show less than 5 Å RMSD are located in the main basin of the SAPPHIRE plot, which means they are similar to representative structures obtained from the μs-MD simulations. The selected visualizations shown for some basins (Figure S 1) show that the

large central basin is composed of variations of the twisted β-hairpin observed also in the CF prediction. Thus, the SAPPHIRE analysis of the μs-MD sampling confirms that the individual chains of the DL model structures are in regions of the conformational space that are accessed frequently by the Aβ42 monomer. The asymmetry of the dimers is evidenced by the lack of pairing between the two chains of a trajectory in the same basins. This can be seen in the plot as the fact that apart from the large, middle basin, there is no repetition of colors in any basin. The high peaks in the cut profile between some basins at the edges of the SAPPHIRE plot imply that very few transitions between the states are observed, therefore meaning some single chains are locked in certain conformations and cannot easily switch to the conformations observed in the main basin.

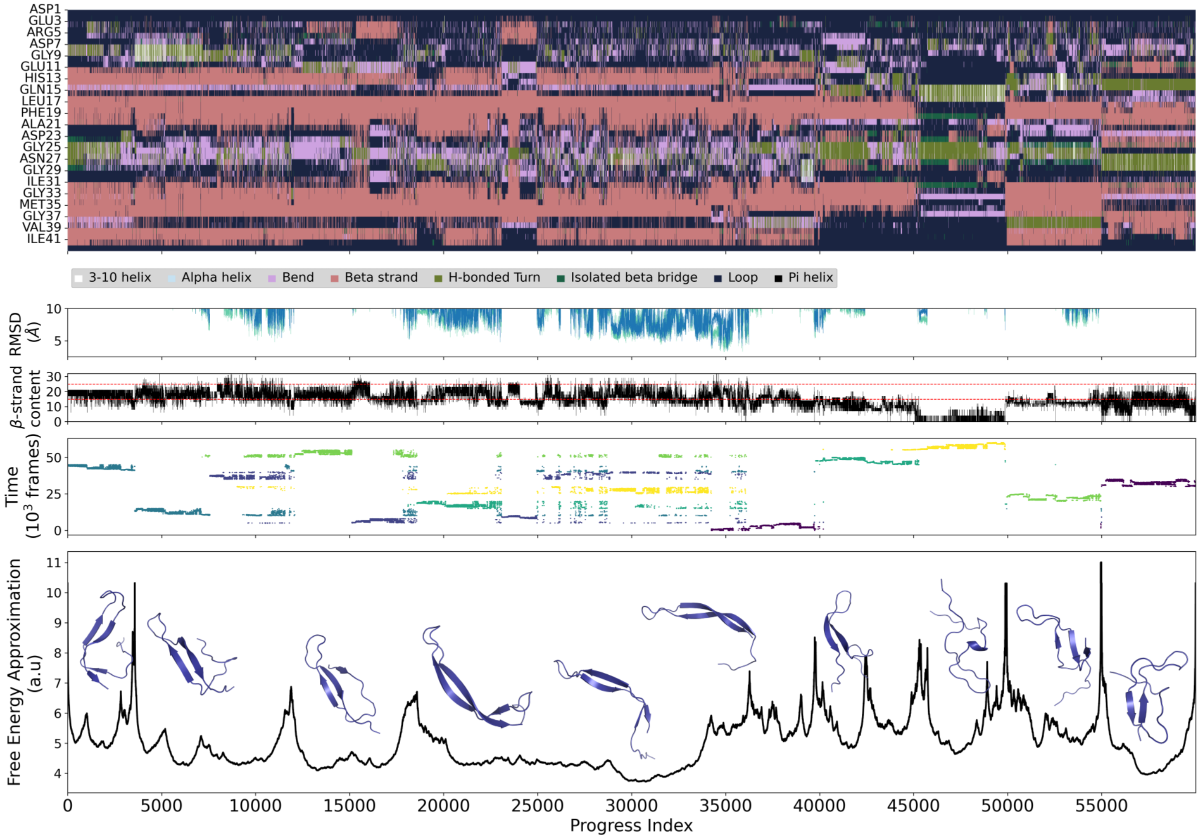

Figure S 1: SAPPHIRE plot of the μs-MD (6 μs) sampling of the Aβ42 dimer.[1] The two chains are treated independently so that the cumulative sampling is 2 x 6 μs from 6 independent runs. (Top) Sequence profile of secondary structure. (Middle) Cα RMSD from the CF (blue) and the AF-M (green) structures. The number of β-strand secondary structure residues is shown (black line) together with two reference values (red horizontal lines). (Bottom) The temporal annotation (dots) illustrates the position of each frame of the trajectory along the cut-based free energy profile. The colors of the dots reflect the six independent runs. The repetition of colors reflects the two chains of each individual trajectory. Cut-based free energy profile (black). The insets show structures sampled from each basin.

Comparison to experimental data. Circular dichroism (CD) spectra provide information on the secondary structure content of (poly)peptides. A caveat is that the CD spectrum represents the ensemble of the molecules in the sample and does not yield residue-level resolution. Still, CD is a valuable tool that can be used to analyze the ns-MD simulations and thus the starting structure selected of the EF-MD simulations.

We use the PDBMD2CD web server[2] for predicting the CD spectra of the snapshots saved along the ns-MD simulations started from the AF-M model. The server generates a set of predicted CD spectra for each of the uploaded MD frames, as well as a representative spectrum. Then the secondary structure content for the ensemble is also calculated. We have analyzed the frames of the AF-M ns-MD simulations, and the largest basins of the SAPPHIRE analysis at the 20% basin which contains also the ns-MD frames. We also predict the spectra for the basins with 21%, 11%, and 5% of frames (Figure 3 of the main text). The predicted spectra are shown in Figure S 2. As mentioned above, unlike experimental CD which is an ensemble spectrum, the predicted PDBMD2CD spectra are given per frame.

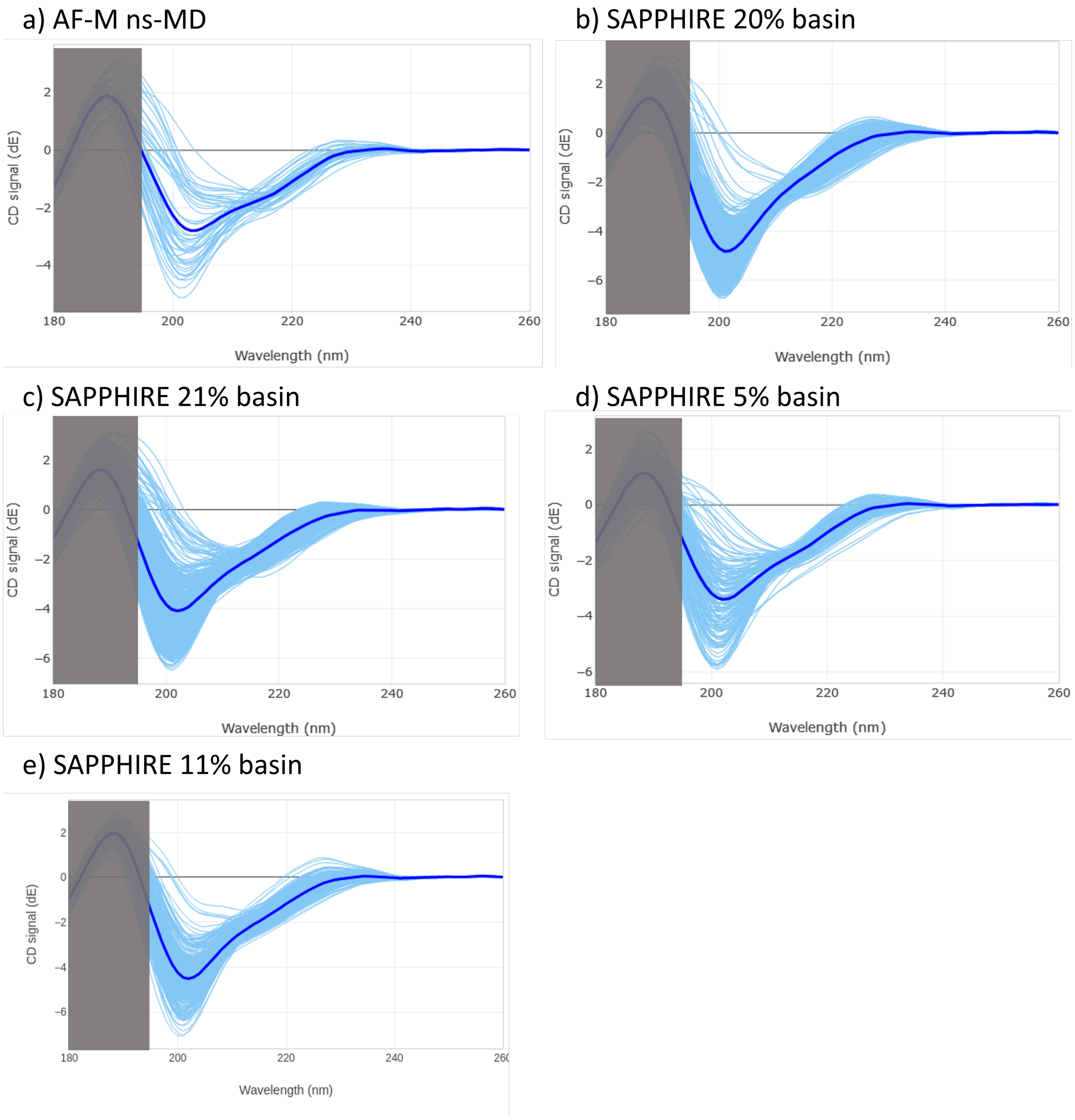

Figure S 2: Predicted CD spectra from PDBMD2CD web server. Each inputted structure generates a spectrum (light blue). Representative spectrum shown in dark blue. A dark rectangle covers the spectra below 195 nm to facilitate comparison with the experimental CD spectra in Figure S 4. (a) ns-MD simulations of AF-M structure. (b)Snapshots from the 20% basin of the μs-MD ensemble shown in Figure 3 of the main text. (c) 21% basin. (d) 5% basin. (e) 11% basin.

The comparison of the predicted (Figure S 2) to the experimental CD spectra of Aβ42

oligomers (Figure S 4) shows an agreement of the predicted spectra to the earlier time points of the experimental spectra. The minimum at around 202 nm is associated with β-sheet structure. Following the time evolution of the experimental spectra at the wavelength around 200 nm it emerges that the calculated spectra are more similar to the first 6 h of 50 μM Aβ42 in 8.6 mM phosphate buffer (Figure S 4A), the first 4 h at 20 mM phosphate buffer (Figure S 4B), and the first 72 h of 25 μM Aβ42 at 50 mM NaCl buffer (Figure S 4C). The lack of α-helical content in both our predicted and the experimental spectra gives us additional confidence on the models generated by deep learning and the behavior of the ns-MD simulations.

The secondary structure content predicted from the CD spectra (Figure S 3) shows that the ns-MD frames have an antiparallel β-sheet content which is slightly higher than that of the μs-MD simulations. Parallel sheet and turn content is more similar to that of the 20% and 21% basins which are close by in the SAPPHIRE plot. The contents differ more to the 11% basin. Helical and loop content are similar in all the four sets of structures analyzed.

a) AF-M ns-MD

**Name of uploaded file/s:**	t_0_0.pdb, t_0_1.pdb, t_0_2...
**Date:**	23-04-18, 14:51
**Number of structures:**	48
**Number of failures:**	0
**Average RMSD from mean:**	0.55
**Name of representative structure:**	t_1_3.pdb

SS Type	Mean and standard deviation (%)
Helix 1	0.0 +/- 0.0
Helix 2	0.07 +/- 0.51
Antiparallel Sheet 1	25.75 +/- 4.14
Antiparallel Sheet 2	22.12 +/- 3.11
Parallel Sheet	3.42 +/- 2.45
Turn	5.98 +/- 2.57
Other	42.66 +/- 7.54

b) SAPPHIRE 20% basin

**Name of uploaded file/s:**	basin_20p_0.pdb, basin_20p_...
**Date:**	23-04-18, 15:02
**Number of structures:**	485
**Number of failures:**	0
**Average RMSD from mean:**	0.42
**Name of representative structure:**	basin_20p_107.pdb

SS Type	Mean and standard deviation (%)
Helix 1	0.0 +/- 0.0
Helix 2	0.6 +/- 1.41
Antiparallel Sheet 1	14.49 +/- 3.42
Antiparallel Sheet 2	18.04 +/- 3.34
Parallel Sheet	3.2 +/- 1.94
Turn	7.77 +/- 3.31
Other	55.91 +/- 6.23

c) SAPPHIRE 21% basin

**Name of uploaded file/s:**	basin_21p_0.pdb, basin_21p_...
**Date:**	23-04-18, 15:31
**Number of structures:**	587
**Number of failures:**	0
**Average RMSD from mean:**	0.47
**Name of representative structure:**	basin_21p_59.pdb

SS Type	Mean and standard deviation (%)
Helix 1	0.01 +/- 0.2
Helix 2	0.63 +/- 1.38
Antiparallel Sheet 1	17.32 +/- 4.39
Antiparallel Sheet 2	15.68 +/- 3.25
Parallel Sheet	2.25 +/- 2.29
Turn	6.98 +/- 3.13
Other	57.13 +/- 6.99

d) SAPPHIRE 5% basin

**Name of uploaded file/s:**	basin_5p_0.pdb, basin_5p_1....
**Date:**	23-04-18, 14:57
**Number of structures:**	159
**Number of failures:**	0
**Average RMSD from mean:**	0.48
**Name of representative structure:**	basin_5p_14.pdb

SS Type	Mean and standard deviation (%)
Helix 1	0.0 +/- 0.0
Helix 2	0.25 +/- 0.91
Antiparallel Sheet 1	19.59 +/- 3.7
Antiparallel Sheet 2	17.65 +/- 3.17
Parallel Sheet	4.98 +/- 1.47
Turn	9.51 +/- 2.83
Other	48.02 +/- 5.89

e) SAPPHIRE 11% basin

**Name of uploaded file/s:**	basin_11p_0.pdb, basin_11p_...
**Date:**	23-05-02, 14:50
**Number of structures:**	350
**Number of failures:**	0
**Average RMSD from mean:**	0.39
**Name of representative structure:**	basin_11p_6.pdb

SS Type	Mean and standard deviation (%)
Helix 1	0.0 +/- 0.0
Helix 2	0.19 +/- 0.81
Antiparallel Sheet 1	18.33 +/- 4.03
Antiparallel Sheet 2	20.01 +/- 4.37
Parallel Sheet	2.53 +/- 2.12
Turn	3.88 +/- 2.14
Other	55.05 +/- 8.28

Figure S 3: Predicted secondary structure content for each set of spectra of Figure S 2.

A) 50 µM Aβ42 in 8.6 mM phosphate buffer. Bartolini, *et. al.*, (2007) *ChemBioChem*

B) 50 µM Aβ42 in 20mM mM phosphate buffer. Vadukul, *et. al.*, (2017) *FEBS Letters*

C) 25 µM Aβ42 in mM mM Tris-HCl 50 mM NaCl buffer. Rangachari, *et. al.*, (2007) *Biochemistry*

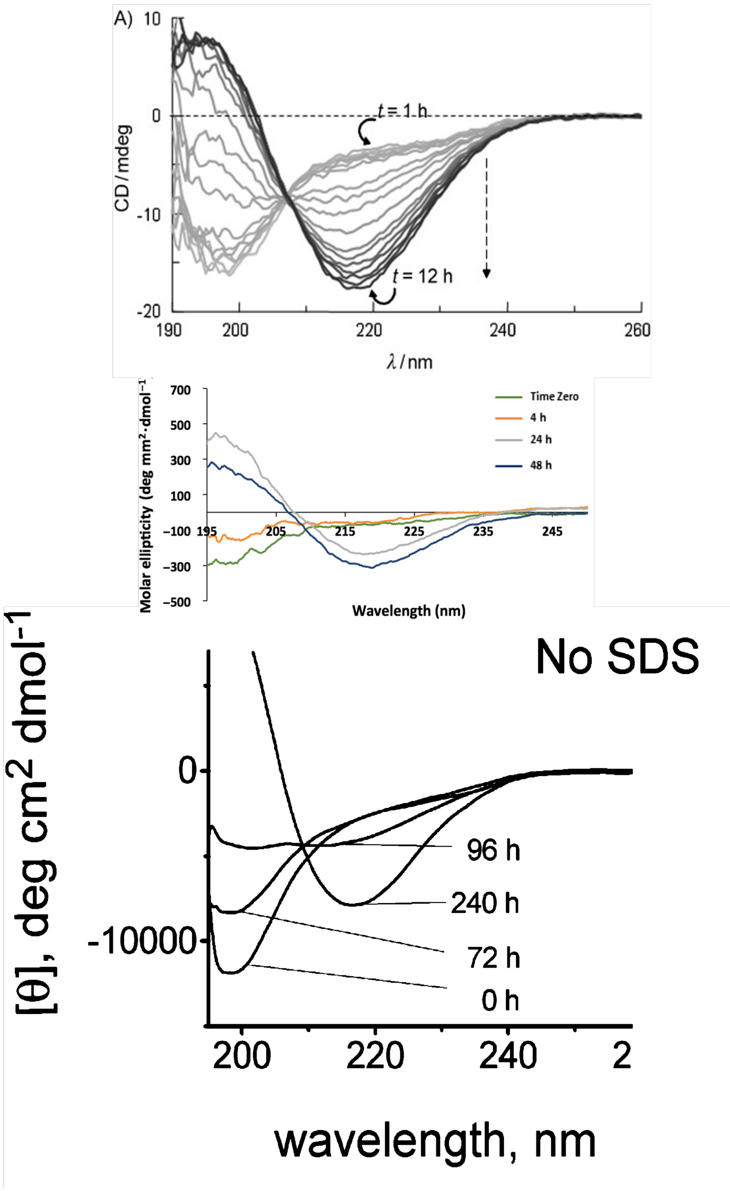

Figure S 4: Experimental CD spectra. (A) CD spectra reprinted in part with permission from Figure 2.A of Bartolini, M.; Bertucci, C.; Bolognesi, M. L.; Cavalli, A.; Melchiorre, C.; Andrisano, V. Insight Into the Kinetic of Amyloid (1–42) Peptide Self-Aggregation: Elucidation of Inhibitors' Mechanism of Action. *ChemBioChem* **2007**, *8*, 2152–2161. Copyright 2007 John Wiley and Sons. Reprinted in part with permission from Figure 3.A of Vadukul, D. M.; Gbajumo, O.; Marshall, K. E.; Serpell, L. C. Amyloidogenicity and toxicity of the reverse and scrambled variants of amyloid- 1-42. *FEBS Letters* **2017**, *591*, 822–830. Copyright **CC BY** license 2017 The Authors. (C) Figure 5.A reproduced from Rangachari, V.; Moore, B. D.; Reed, D. K.; Sonoda, L. K.; Bridges, A. W.; Conboy, E.; Hartigan, D.; Rosenberry, T. L. Amyloid-β (1- 42) rapidly forms protofibrils and oligomers by distinct pathways in low concentrations of sodium dodecylsulfate. *Biochemistry* **2007**, *46*, 12451–12462. Copyright 2007 American Chemical Society.

Effect of an oscillating electric field on dimeric Aβ42.

Supplementary movies 1 to 4 show the evolution of a single EF-MD trajectory for each field strength of 0, 10, 100, or 200 mV/nm, respectively. The two Aβ42 chains are colored blue and red, respectively, and the N termini are labelled by a sphere.

The secondary structure for all EF-MD trajectories shows that for the 0 mV/nm (no potential, Figure S 5) and 10 mV/nm (low potential, Figure S 6) simulations, the secondary structure is very stable and remains mostly as β strands. The RMSD also remains low for both conditions.

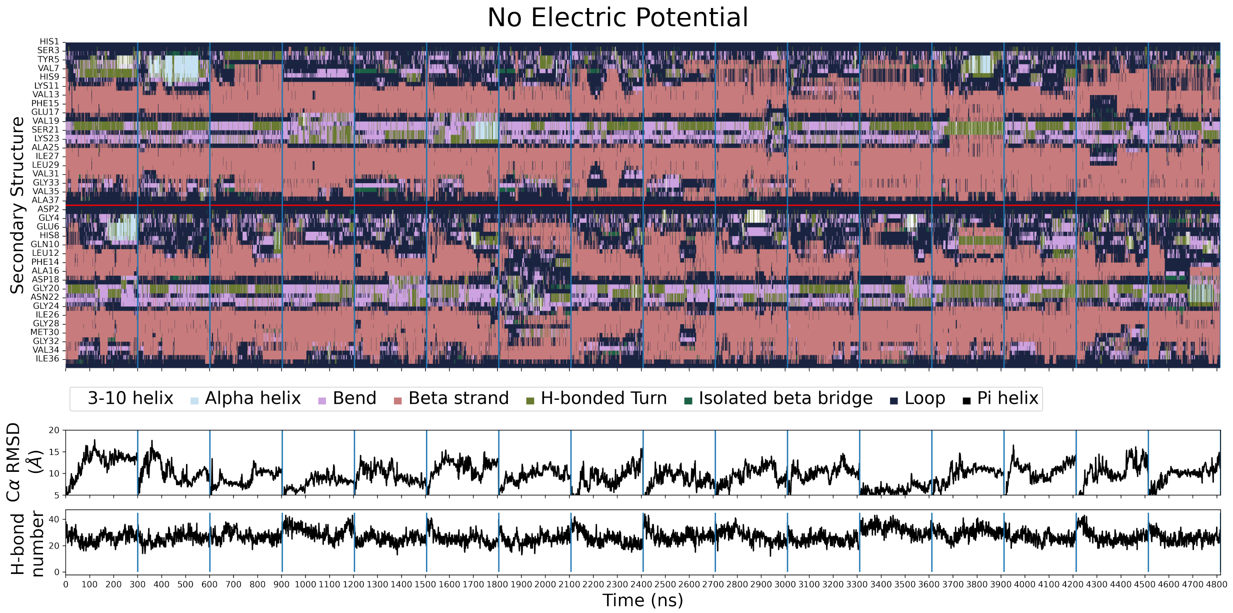

Figure S 5: Structural stability of Aβ42 in the 16 independent MD runs without electric field (EF-MD 0 mV/nm). (Top) Secondary structure content calculated by dssp. (Bottom) RMSD with respect to the initial AF-M structure and number of solute hydrogen bonds, i.e., sum of intra-peptide and inter-peptide hydrogen bonds.

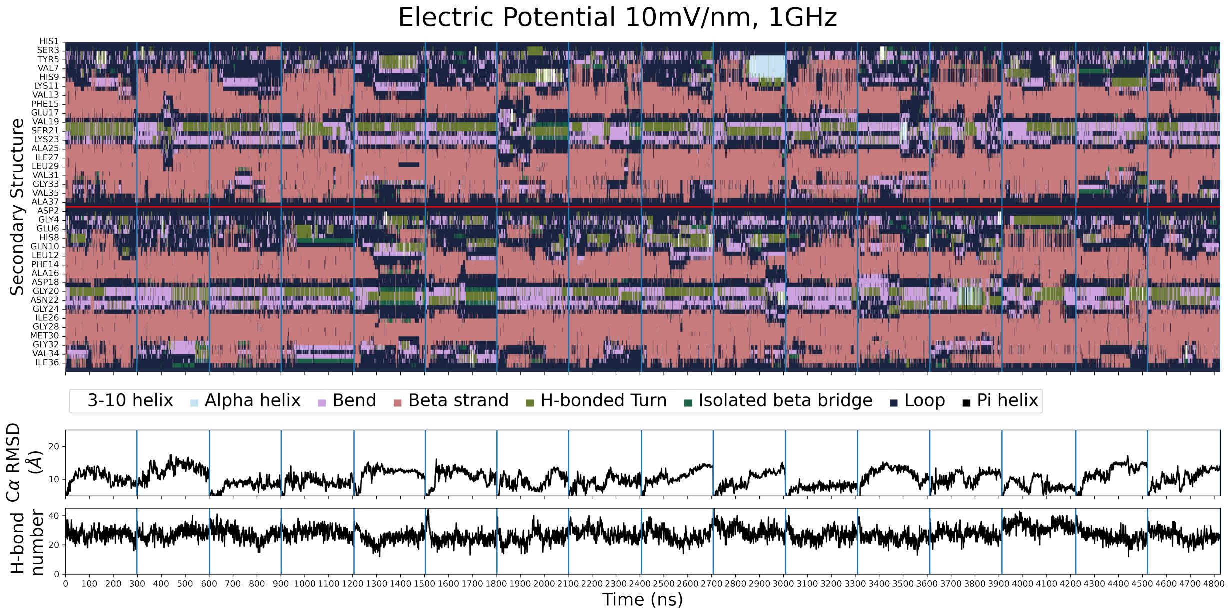

Figure S 6: The same as Figure S 5 for a field strength of 10 mV/nm.

The 100 mV/nm EF-MD trajectories (Figure S7) show an increasing decay of the β strands. Small α-helical segments appear for short times (around 10 ns) and then decay to bends or loops. The 200 mV/nm EF-MD (Figure S 8) shows an almost immediate disruption of the β-strand secondary structure and an increase in loops and turns. Helical structures are transient.

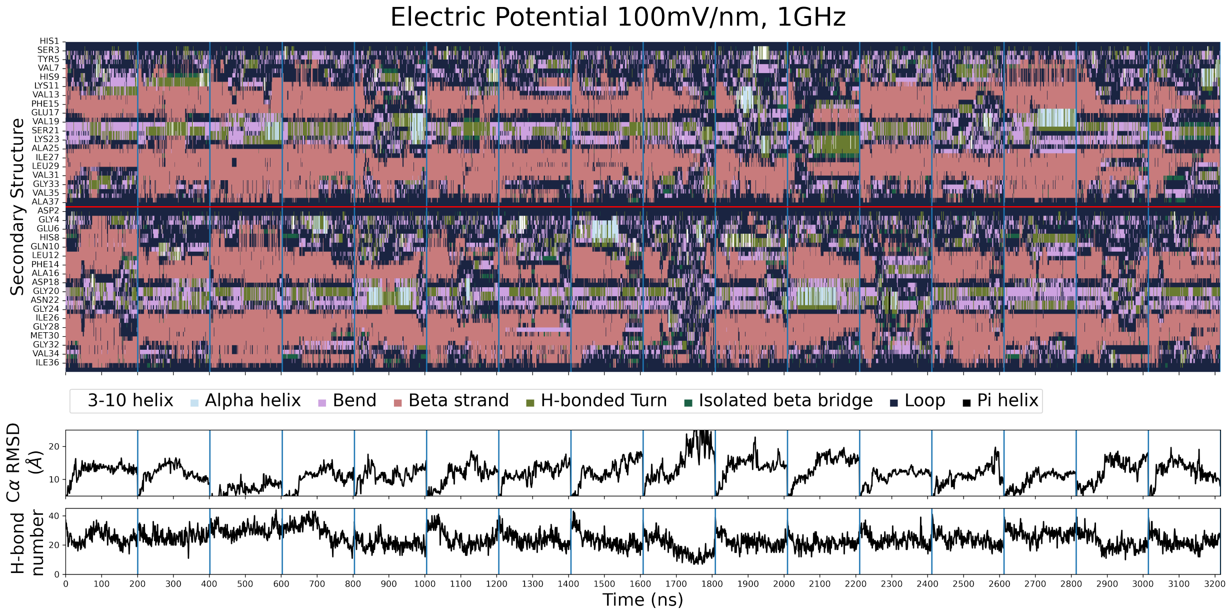

Figure S 7: The same as Figure S 5 for a field strength of 100 mV/nm.

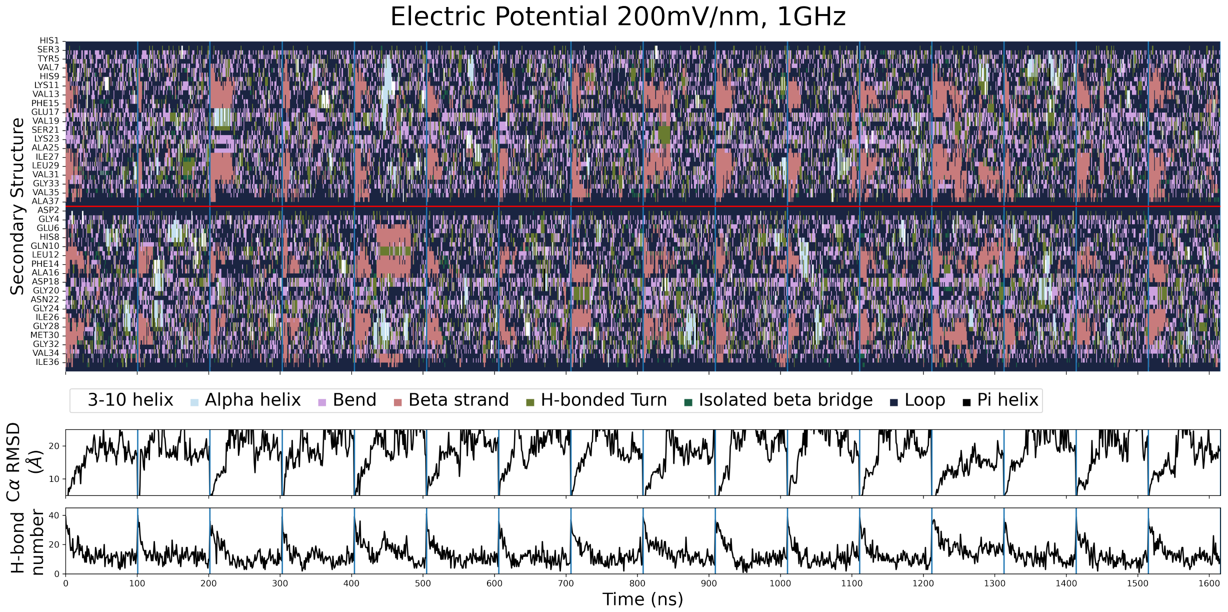

Figure S 8: The same as Figure S 5 for a field strength of 200 mV/nm.

Effect of an oscillating electric field on the leucine zipper.

A control simulation of the HY5 leucine zipper shows a much stronger stability of the helical dimer under the influence of the electric field of the same oscillating frequency (1 GHz). No change in secondary structure content is observed during the 200 ns of simulation for the leucine zipper without electric field or at 100 mV/nm (Figures S 9 and S 10). At 200 mV/nm a clear time dependent decay can be observed (Figure S 11) in all runs.

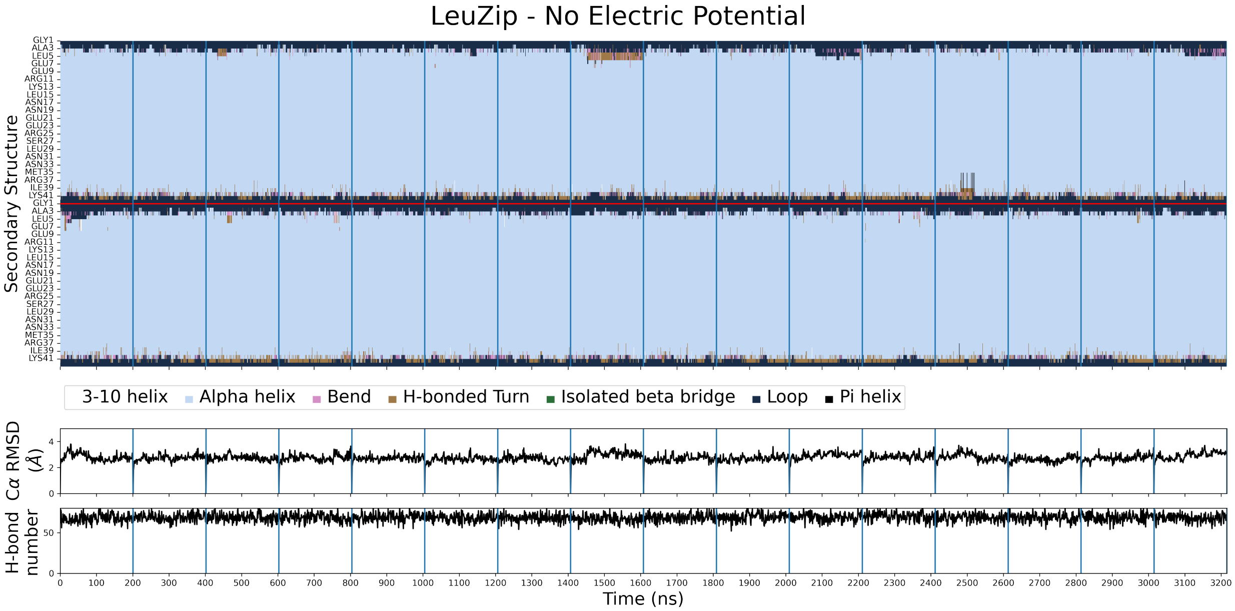

Figure S 9: Structural stability of the leucine zipper in 16 independent MD runs without electric field. (Top) Secondary structure content calculated by dssp. (Bottom) RMSD with respect to the crystal structure (PDB code: 2OQQ) and number of solute hydrogen bonds, i.e., sum of intra-peptide and inter-peptide hydrogen bonds.

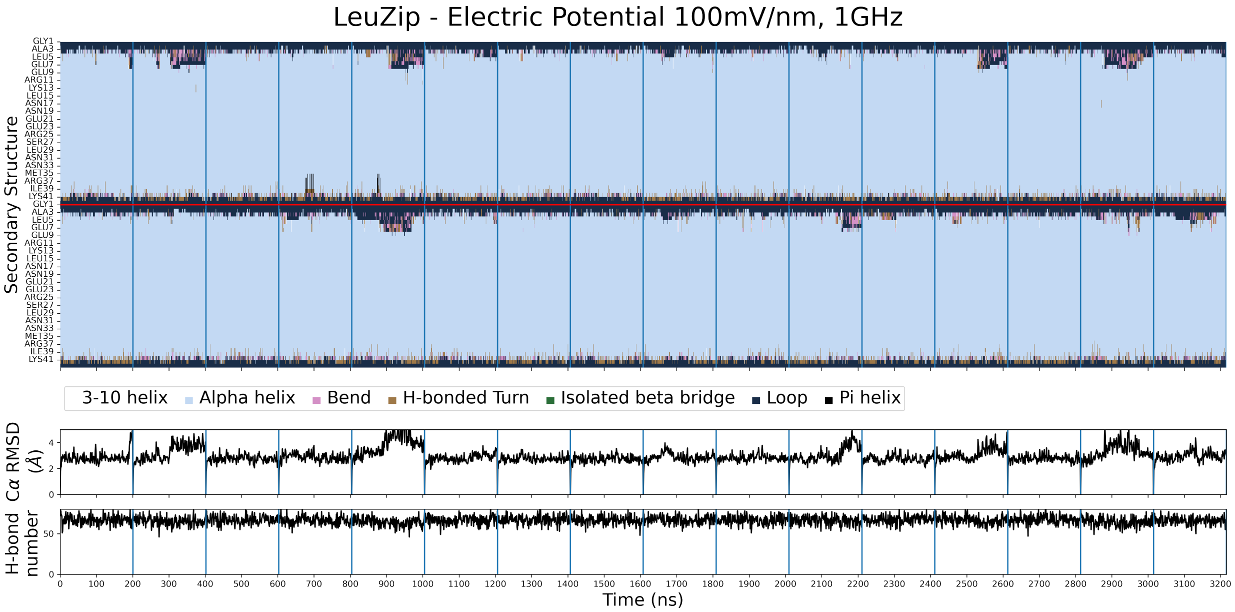

Figure S 10: The same as Figure S 9 for a field strength of 100 mV/nm.

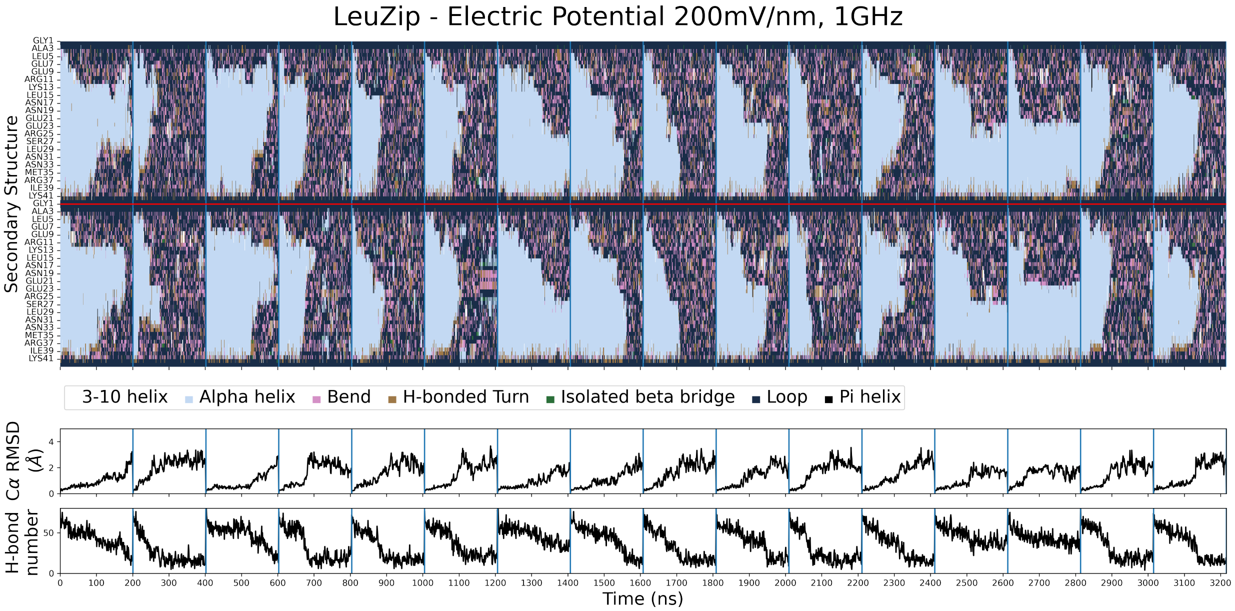

Figure S 11: The same as Figure S 9 for a field strength of 200 mV/nm.

References

(1) Fatafta, H.; Khaled, M.; Owen, M. C.; Sayyed-Ahmad, A.; Strodel, B. Amyloid-β peptide dimers undergo a random coil to β-sheet transition in the aqueous phase but not at the neuronal membrane. *Proceedings of the National Academy of Sciences of the United States of America* **2021**, *118*, e2106210118.

(2) Drew, E. D.; Janes, R. W. PDBMD2CD: providing predicted protein circular dichroism spectra from multiple molecular dynamics-generated protein structures. *Nucleic Acids Research* **2020**, *48*, W17–W24.

(3) Bartolini, M.; Bertucci, C.; Bolognesi, M. L.; Cavalli, A.; Melchiorre, C.; Andrisano, V. Insight Into the Kinetic of Amyloid (1–42) Peptide Self-Aggregation: Elucidation of Inhibitors' Mechanism of Action. *ChemBioChem* **2007**, *8*, 2152–2161.

(4) Rangachari, V.; Moore, B. D.; Reed, D. K.; Sonoda, L. K.; Bridges, A. W.; Conboy, E.; Hartigan, D.; Rosenberry, T. L. Amyloid-β (1- 42) rapidly forms protofibrils and oligomers by distinct pathways in low concentrations of sodium dodecylsulfate. *Biochemistry* **2007**, *46*, 12451–12462.
